# Transcriptional profiling and biomarker identification reveal tissue specific effects of expanded ataxin-3 in a spinocerebellar ataxia type 3 mouse model

**DOI:** 10.1186/s13024-018-0261-9

**Published:** 2018-06-22

**Authors:** Lodewijk J. A. Toonen, Maurice Overzier, Melvin M. Evers, Leticia G. Leon, Sander A. J. van der Zeeuw, Hailiang Mei, Szymon M. Kielbasa, Jelle J. Goeman, Kristina M. Hettne, Olafur Th. Magnusson, Marion Poirel, Alexandre Seyer, Peter A. C. ‘t Hoen, Willeke M. C. van Roon-Mom

**Affiliations:** 1 Department of Human Genetics, Leiden University Medical Center, 2300 RC Leiden, The Netherlands; 2 Department of Research & Development, uniQure, Amsterdam, The Netherlands; 3 Cancer Pharmacology Lab, University of Pisa, Ospedale di Cisanello, Edificio 6 via Paradisa, 2, 56124 Pisa, Italy; 4 Sequencing Analysis Support Core, Leiden University Medical Center, 2300 RC Leiden, The Netherlands; 5 Department of Biomedical Data Sciences, Leiden University Medical Center, 2300 RC Leiden, The Netherlands; 6 deCODE genetics/Amgen, Sturlugata 8, 101 Reykjavik, Iceland; 7 MedDAY Pharmaceuticals, Paris, France; 8 Centre for Molecular and Biomolecular Informatics, Radboud Institute for Molecular Life Sciences, Radboud University Medical Center, 6500 HB, Nijmegen, The Netherlands

**Keywords:** Spinocerebellar ataxia type 3, Mouse model, RNA sequencing, Metabolomics

## Abstract

**Background:**

Spinocerebellar ataxia type 3 (SCA3) is a progressive neurodegenerative disorder caused by expansion of the polyglutamine repeat in the ataxin-3 protein. Expression of mutant ataxin-3 is known to result in transcriptional dysregulation, which can contribute to the cellular toxicity and neurodegeneration. Since the exact causative mechanisms underlying this process have not been fully elucidated, gene expression analyses in brains of transgenic SCA3 mouse models may provide useful insights.

**Methods:**

Here we characterised the MJD84.2 SCA3 mouse model expressing the mutant human ataxin-3 gene using a multi-omics approach on brain and blood. Gene expression changes in brainstem, cerebellum, striatum and cortex were used to study pathological changes in brain, while blood gene expression and metabolites/lipids levels were examined as potential biomarkers for disease.

**Results:**

Despite normal motor performance at 17.5 months of age, transcriptional changes in brain tissue of the SCA3 mice were observed. Most transcriptional changes occurred in brainstem and striatum, whilst cerebellum and cortex were only modestly affected. The most significantly altered genes in SCA3 mouse brain were *Tmc3*, *Zfp488*, *Car2*, and *Chdh*. Based on the transcriptional changes, α-adrenergic and CREB pathways were most consistently altered for combined analysis of the four brain regions. When examining individual brain regions, axon guidance and synaptic transmission pathways were most strongly altered in striatum, whilst brainstem presented with strongest alterations in the pi-3 k cascade and cholesterol biosynthesis pathways. Similar to other neurodegenerative diseases, reduced levels of tryptophan and increased levels of ceramides, di- and triglycerides were observed in SCA3 mouse blood.

**Conclusions:**

The observed transcriptional changes in SCA3 mouse brain reveal parallels with previous reported neuropathology in patients, but also shows brain region specific effects as well as involvement of adrenergic signalling and CREB pathway changes in SCA3. Importantly, the transcriptional changes occur prior to onset of motor- and coordination deficits.

**Electronic supplementary material:**

The online version of this article (10.1186/s13024-018-0261-9) contains supplementary material, which is available to authorized users.

## Background

Spinocerebellar ataxia type 3 (SCA3), also known as Machado Joseph Disease (MJD), is a progressive neurodegenerative disorder, with symptoms usually presenting around midlife. SCA3 is the most common of the dominantly inherited ataxias and is caused by a CAG repeat expansion in the *ATXN3* gene [1]. The CAG repeat is translated into a polyglutamine (polyQ) stretch in the ataxin-3 protein, which upon mutational expansion to 56–84 glutamines results in a gain of toxic protein function [2]. This protein toxicity mostly shows its effects in the brain, and neuronal loss in SCA3 has been reported predominantly in the brainstem, cerebellum (spinocerebellar pathways and dentate nucleus), striatum, thalamus, substantia nigra and pontine nuclei [3]. Over time, the neuronal loss causes clinical symptoms in SCA3 patients such as progressive ataxia, dystonia, spasticity, and various other symptoms (reviewed in [1]).

The molecular mechanisms of mutant ataxin-3 toxicity have been the subject of extensive research, and a range of cellular changes have been suggested to contribute to toxicity. These include aggregation and nuclear localisation of expanded ataxin-3 protein [4, 5], impaired protein degradation [6], mitochondrial dysfunction [7] and transcriptional deregulation [8]. Transcriptional deregulation may arise due to sequestration of transcription factors such as TATA-box binding protein [9] and CREB binding protein (CBP) [10] into the polyQ aggregates, thereby interfering with their function. Previous gene expression studies have identified altered inflammatory processes, cell signalling and cell surface associated genes in cell and conditional animal models of SCA3 [8, 11, 12]. Despite these recent advances in SCA3 pathogenicity, it is currently still not fully elucidated which molecular mechanisms are altered in response to mutant ataxin-3. For this reason, it is useful to examine genetic mouse models of SCA3 for transcriptional changes that occur in different regions of the brain to infer causative disease mechanisms [13].

Apart from gaining insight into disease mechanisms, transcriptional changes may also be potentially useful as biomarkers to track disease progression in SCA3. Since it is not possible to study longitudinal gene expression changes in human brain tissue, it is useful to establish potential transcriptional changes in peripheral tissues such as blood. In addition, metabolite and lipid changes in blood can also be used as easily obtainable biomarkers, and can potentially be used to track disease progression [14]. Previous research by our group has shown that there are common gene expression signatures in blood and brain of patients with Huntington disease [15]. Since patient material is not readily available, genetic SCA3 mouse models are a good starting point to identify such potential disease biomarkers.

In this study, we set out to identify the molecular mechanisms involved in SCA3 pathology. Current next-generation sequencing techniques provide an attractive means to objectively study the transcriptome and allow for very sensitive and accurate assessment of changes in gene expression. As such, we performed RNA sequencing of brain and blood from the hemizygous MJD84.2 mouse model of SCA3, which ubiquitously expresses the full human *ATXN3* gene with 76–77 CAGs [16] and gene expression analysis was performed in 4 different regions of the brain. Additionally, blood samples from the mice were subjected to RNA sequencing and serum was used for metabolomic and lipidomic analysis to identify potential biomarkers capable of tracking disease progression.

We found that the MJD84.2 mice presented with reduced bodyweight compared to wild-type, but did not develop motor symptoms even at 17.5 months of age. Gene expression changes in blood were also not pronounced, with pathway analysis suggesting respiratory electron transport and mitochondrial function to be affected. In parallel to other neurodegenerative disorders, further metabolomic and lipidomic analyses of blood revealed decreased tryptophan and increased levels of a di- and triglycerides and ceramides in SCA3 mice. In contrast to blood, transcriptional changes were readily detected in brain, with the highest number of differentially expressed genes in brainstem and striatum. Somewhat surprisingly, the cerebellum was affected to a smaller extent compared to these two brain regions. The main deregulated pathways in brain were cellular signalling pathways (α-adrenergic and CREB signalling) and pathways related to synaptic transmission. This study hence provides additional evidence for affected CREB signalling in SCA3 and suggests affected neurotransmission pathways, particularly in striatum.

## Methods

### SCA3 mice and tissue sampling

MJD84.2 transgenic SCA3 mice [16] and wild-type C57BL/6 mice were obtained from Jackson Laboratories (Bar Harbor, Maine, USA). All animal experiments were carried out in accordance with European Communities Council Directive 2010/63/EU and were approved by the Leiden University animal ethical committee. Breeding was performed by crossing hemizygous SCA3 mice with wild-types. ATXN3 CAG repeat lengths were verified for each mouse through gene fragment analysis, using human specific primers (Additional file 1: Table S1) flanking the CAG repeat similar to described previously [17]. Human *ATXN3* repeat lengths were 76 or 77 for all transgenic mice. Only male mice were used, and a total of 8 transgenic and 8 wild-type mice were included in the experiment (Table 1), though 2 transgenic mice did not survive to the end of the study. Mice were group housed in individually ventilated cages with food and drinking water available ad libitum. Blood samples for metabolomic analyses were obtained at 4, 12 and 16 months of age from 4 wild-type and 4 SCA3 mice. Animals were fasted 4 h prior to obtaining 200 μl blood through tail cut and collection in heparin lithium tubes. Tubes were immediately spun down at 18,000 x g and the supernatant (plasma) was stored at − 80 °C. For RNA sequencing, 200 μl of blood was obtained by tail cut at 9 months and 17.5 months of age. Blood samples for RNA sequencing were collected in RNAprotect animal blood tubes (Qiagen) following manufacturer’s instructions, stored overnight at 4 °C and subsequently frozen at − 80 °C until RNA isolation. At 17.5 months of age, mice were sacrificed and brainstem, cerebellum, striatum and cortex were dissected, snap-frozen in liquid nitrogen and stored at − 80 °C.

**Table 1:** RNA sequencing and metabolomic/lipidomic sample overview

Analysis	Tissue	Wild-type mice	SCA3 mice
RNA-seq	brainstem	8	6
RNA-seq	cerebellum	7	6
RNA-seq	cortex	7	6
RNA-seq	striatum	8	5
RNA-seq	blood (9 and 17.5 months)	6	5
Metabolomics	plasma (4 and 16 months)	4	4
Lipidomics	plasma (4 and 16 months)	4	4 (4 months), 3 (16 months)

### Behavioural testing

To assess the motor phenotype and coordination of the mice, a beamwalk test was performed. The beamwalk balance test consisted of 2 boxes (20 × 20 × 20 cm) elevated at 53 cm height and connected by a plastic cylindrical bar of ø 10 mm or ø 30 mm and 80 cm long. Mice were placed in the transparent elevated box and crossed the bar to an enclosed dark box. The average latency to cross from 3 trials per testing day is reported. The beamwalk test was performed when the mice were 4, 6, 7.5, 9 and 12 months of age.

### Metabolite profiling in plasma

Analysis of the plasma samples was performed by Profilomics (Gif-sur-Yvette, France). For extraction of metabolites, 15 μL plasma sample was treated with 60 μL of methanol with a mixture of internal standards. Protein was precipitated at 4 °C, centrifuged and supernatants were dried under nitrogen. Samples were then resuspended in ammonium carbonate 10 mM pH 10.5/AcN 40:60 (*v*/v). Chromatography settings for LC-HRMS were followed as outlined by Boudah et al.*..* [18]. Plasma extracts were separated on a HTC PAL-system (CTC Analytics AG, Zwingen, Switzerland) coupled with a Transcend 1250 liquid chromatographic system (ThermoFisher Scientific, Les Ulis, France) using an aSequant ZICpHILIC 5 μm, 2.1 × 150 mm at 15 °C (Merck, Darmstadt, Germany). After injecting 10 μL of sample, the column effluent was directly introduced into the heated Electrospray (HESI) source of a Q-Exactive mass spectrometer (Thermo Scientific, San Jose, CA) and analysis was performed in both ionization modes. Identification of molecules was performed using TraceFinder3.1 software (ThermoFisher Scientific, Les Ulis, France). The dataset was filtered and cleaned based on quality control samples as described by Dunn et al [19].

### Lipid profiling in plasma

Analysis of lipids in plasma was performed on identical samples as described for the metabolite analysis. Lipid analyses were performed at Profilomics (Gif-sur-Yvette, France), in accordance with previously described methods [20]. In brief, 50 μL of plasma was added to 245 μL of CHCl3/MeOH 1:1 (*v*/v) and 5 μL of internal standard mixture. Extraction was performed after 2 h at 4 °C and centrifugation at 15,000×g for 10 min at 4 °C. The upper phase (aqueous phase), containing ganglioside species and several lysophospholipids, was transferred and dried under a stream of nitrogen. The protein interphase was discarded and the lower rich-lipid phase (organic phase) was pooled with the dried upper phase. Samples were then reconstituted in 50 μl CHCl3/MeOH 1:1, vortexed for 30 s, sonicated for 60 s and diluted 100 times in MeOH/IPA/H2O 65:35:5 (v/v/v) before injection. Similar to metabolite detection, plasma total lipid extracts were separated on HTC PAL-system (CTC Analytics AG) coupled with a Transcend 1250 liquid chromatographic system (ThermoFisher Scientific) using a kinetex C8 2.6 μm 2.1 × 150 mm column (Phenomenex, Sydney, NSW, Australia). Mass spectrometry was performed similar as for the metabolites and data processing was done as previously described [20].

### RNA isolation

After thawing, filled blood tubes were incubated for 4 h at 25 °C to ensure proper cell lysis. Isolation of RNA was subsequently performed using the RNeasy protect animal blood kit (Qiagen, Hilden, Germany) according to manufacturer’s instructions for total RNA isolation including DNAse treatment, resulting in isolation of RNA molecules longer than approximately 200 nucleotides. Reduction of alpha and beta globin mRNA was performed on RNA samples using the GLOBINclear magnetic bead kit for mouse/rat (Qiagen) following manufacturer’s instructions.

For isolation of RNA from brain tissue, approximately 30 mg of tissue was transferred to next advance pink bead tubes (Next Advance, Averill Park, US) containing 500 μl Trizol (Ambion, Thermo Fisher scientific, Waltham, MA, USA). Tissue was homogenised in a bullet blender BBX24 (Next Advance) for 3 min on setting 8. A total of 100 μl chloroform was added and samples were spun down at 10,000 x g for 15 min. The aqueous phase contining the RNA was removed and an equal volume of 70% ethanol was added. RNA purification was then performed using the PureLink RNA mini kit (Thermo Fisher scientific) in accordance with the manufacturer’s protocol using provided RNA columns and a 15 min DNase step. RNA was eluted in 80 μl nuclease free water. Concentration and purity of RNA was measured using Nanodrop spectrophotometry and RNA was stored at − 80 °C.

### RNA sequencing

Library preparation and RNA sequencing was performed at deCode Genetics (Reykjavik, Iceland). The quality of RNA was assessed with the LabChip GX using the 96-well RNA kit (Perkin Elmer). Approximately 1 μg of total RNA was used as starting material, and the average RIN values were 7.7 (SD ± 0.5) for brain tissue and 6.8 (SD ± 0.9) for blood. Non strand-specific sample preparation was performed using the TruSeq Poly-A v2 kit (Illumina, San Diego, USA) following manufacturer’s instructions. In brief, mRNA was captured using magnetic poly-T oligo-attached magnetic beads, RNA molecules were fragmented, and cDNA synthesis was performed using SuperScript II (Invitrogen, Carlsbad CA, USA) with random hexamer primers. Subsequently, 2nd strand cDNA synthesis was performed in conjunction with RNAse-H treatment. End repair was performed to generate blunt ends and 3′ adenylation was performed, followed by ligation of indexing adapters to the ds-cDNA. PCR was performed to amplify the fragments. Quality of sequencing libraries was determined through pool sequencing on a MiSeq instrument (Illumina) to assess insert size, sample diversity and optimize cluster densities. Pooled samples (4 samples/pool) were clustered on paired-end (PE) flowcells (1 pool per lane) using a cBot instrument (Illumina). The sequencing was performed using a HiSeq 2500 with v4 SBS sequencing kits (read lengths 2 × 125 cycles). Primary processing and base calling was performed with Illumina’s HCS and RTA. Demultiplexing and generation of FASTQ files was done with Illumina scripts (bcl2fastq v1.8). The FASTQ files for the mouse brain RNA can be found in the GEO repository, accession GSE107958 and blood samples are listed under accession GSE108069.

### Sequencing data processing

Analysis of sequencing data was performed using the BIOPET Gentrap in-house pipeline (http://biopet-docs.readthedocs.io/en/v0.7.0/pipelines/gentrap/) The fastqc toolkit (v0.11.2) was used to evaluate sequencing quality (http://www.bioinformatics.babraham.ac.uk/projects/fastqc/). Sickle (v1.33 with default settings) and Cutadapt (v1.10, with default settings except for “-m 20”) were used to clean up reads. Cleaned reads were aligned to the mouse reference genome build 10 (GRCm38/mm10) using STAR aligner version 2.3.0e [21]. The non-default settings used by STAR are “--outFilterMultimapNmax 1 –outFilterMismatchNmax 10 –outSJfilterReads Unique”. Average number of reads was 84 million (SD ± 18 million). On average, 66% of reads were aligned to known genes. Gene raw read counts are generated using *HTSeq* (v0.6.1) with the Refseq gene annotation extracted from UCSC on 11–09-2015. The non-default settings used by *HTSeq are “*--format bam –stranded no”. Gene expression analysis was performed using edgeR (v 3.14.0) [22]. The normalization was performed using the trimmed mean of M-values (TMM) normalization method [23].

### Differential gene expression and statistical analysis

Analysis of gene expression was performed on genes exceeding an average 4 counts per million (CPM) across all samples. Principle component analysis (PCA) was performed to confirm sample consistency (i.e. clustering per brain region). Additionally, correlation between genotype and GC percentage or 5′ - 3′ ratios was assessed for potential confounding effects. Differential gene expression was performed using the generalized linear model (GLM) likelihood ratio test functionality of edgeR [22]. Analyses were performed for the 4 brain regions separately, but also as a combined dataset, which is termed “brain data combined” data throughout the manuscript. For this combined analysis of all brain regions, we modelled the effect of strain and tissue (brain region) and the interaction between them to allow detection of strain effects that were either present in all brain regions or tissue-specific. For this, a design matrix was created with the function model.matrix(~ Tissue * Strain) and dispersion was estimated accounting for this design. A general linear model was fit using the glmFit function, and likelihood ratio test then performed with glmLRT on the combination of coefficients for Strain and the interaction term Tissue*Strain. The null hypothesis is that the gene shows no differential expression in any brain region. This analysis is powerful for finding genes with weak effects in several brain regions, but does not allow inference of differential expression in any specific brain regions. For differential gene expression analysis between SCA3 and wildtype mice within individual brain regions, one coefficient was assigned to each group using model.matrix(~ 0 + group). Likelihood ratiotest was then performed using glmLRT function with contrast argument to allow pairwise genotype comparison for each brain region. Analysis of differential gene expression for blood was performed similar to brain, but due to observation of a confounding influence, GC-content correction was first performed using the conditional quantile normalization (CQN) package as previously described [24]. The GC-content correction offset obtained from CQN was then included when estimating dispersion in edgeR. The two time points (9 and 17.5 months) were included as contrasts for the likelihood ratiotest. Genes with a false discovery rate (FDR, Benjamini-Hochberg) below 0.05 were considered significant. Plots were generated using ggplot2 package [25] or graphpad Prism 7. Analysis of the metabolites and lipids in blood was performed using a Welch’s t-test without multiple testing correction (due to 4 vs 4 sample size), and nominal *p*-values < 0.05 were considered significant.

### Functional annotation of gene sets and pathway analysis

For identification of functional processes, sets of genes with a FDR of < 0.05 were used, for each individual brain region and also for all brain regions combined. This led to inclusion of 585 genes from all brain regions combined, 195 genes for brainstem and 824 genes from striatum. Cerebellum and cortex did not present with enough differentially expressed genes to perform pathway analysis. Pathway analysis and exploration of metabolite-phenotype links was performed using Ingenuity Pathway Analysis (IPA) and the Euretos Knowledge Platform (EKP) [26]. Euretos allows for semantic search for biologically interesting connections between genes, proteins, metabolites and drugs based on an underlying database of 176 integrated data sources (January 2017) [27]. Pathway analysis was performed by the use of the Fisher exact test for gene set enrichment. Overlapping significantly altered pathways between the Euretos and Ingenuity analysis were considered as the most reliable signal, and are hence listed as top overrepresented pathways.

### Validation with RT-qPCR

RNA sequencing results were validated on the same RNA samples using qPCR. cDNA synthesis was performed using oligo-dT primers for brain and random hexamer primers for blood RNA, with the Transcriptor First Strand cDNA Synthesis Kit (Roche, Mannheim, Germany) similar to described previously [28], but using an incubation step of 60 min at 50 °C. qPCR was performed with SensiMix SYBR & Fluorescein Kit (Bioline, Taunton, USA) similar to previously described [28], using 3 μl of 5× diluted cDNA for brain samples and 3 μl of 15× diluted cDNA for blood. Mouse reference genes used were β-actin (*Actb*), Hypoxanthine-guanine phosophoribosyltransferase (*Hprt*), and Ribosomal Protein L22 (*Rpl22*) for brain tissue and *Actb*, vinculin (*Vcl*) and *Hprt* for blood (Additional file 1: Table S1). Primers were designed with Primer3 software [29] and PCR efficiencies and expression values (N0) were determined using LinRegPCR 2014.0 ^19^. Transcript level expression was then divided by the geometric mean of the 3 reference genes expression [30]. Statistical tests were performed in graphpad (7.0) using the two-stage linear step-up multiple testing procedure of Benjamini, Krieger and Yekutieli, with Q = 5% and without assuming a consistent SD.

### Western blotting

Protein isolation and western blotting of mouse brain tissue was performed following standard protocols. In brief, brain tissue was homogenized in RIPA buffer using a bullet blender BBX24 (Next Advance, Averill Park, US). Protein concentration was determined using the bicinchoninic acid kit (Thermo Fisher Scientific). A total of 30 μg protein was boiled for 5 min with 4× Laemmli sample buffer and separated on 10% Tris-glycine precast gel (Biorad, Veenendaal, the Netherlands) and transferred to a nitrocellulose membrane. Membranes were blocked in 5% low fat milk and incubated overnight at 4 °C with primary antibodies: rabbit anti-carbonic anhydrase 2 (car2) 1:2000 (Novus Biologicals, Littleton, CO, USA), rabbit anti-psat1 1:1000 (Novus Biologicals) and as loading control mouse anti-β-actin 1:5000 (Abcam, Cambridge, UK). Detection was performed using secondary antibodies IRDye 680RD and 800CW (LI-COR Biosciences, Lincoln, USA) 1:5000, and membranes were imaged using Odyssey infrared imaging system (LI-COR). Quantification was performed with Odyssey software version 3.0 (Licor) using the integrated intensity method. Intensity of car2 and psat1 protein bands were divided by the β-actin intensity to correct for protein loading.

## Results

### SCA3 mice do not present with overt motor symptoms at 17 months of age

**Fig. 1:**
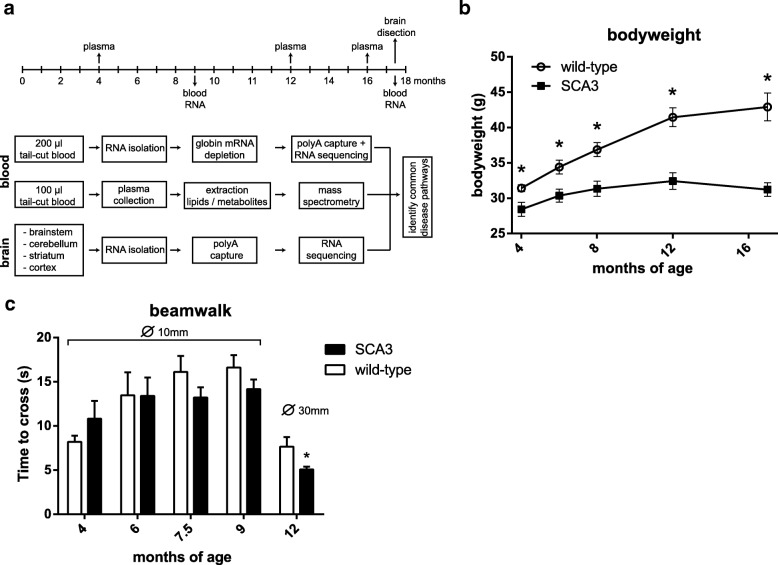
Experimental design and behavioural testing in SCA3 mice. **a** MJD84.2 hemizygous mice were used as a model for SCA3. At indicated time points, plasma was collected for metabolic and lipidomic analyses, and whole blood was collected for RNA sequencing purposes. At 17.5 months of age, mice were sacrificed and 4 brain regions were isolated for RNA sequencing. **b** SCA3 mice show significantly lower bodyweight compared to wild-type mice. **c** The beamwalk balance test shows identical performance in coordination/balance performance of SCA3 and wild-type mice, apart from a better performance of SCA3 mice at 12 months of age. Depicted data represents 8 wild-type vs 8 SCA3 mice. Shown is mean +/− SEM, * = p < 0.05 using multiple t-test

The MJD84.2 mouse model ubiquitously expresses full-length mutant human ataxin-3 with 76–77 glutamines, under control of the human ataxin-3 promoter. During a 17.5 month period, the behavioural phenotype of the mice was assessed using motor tests, and blood was collected for assessment of biomarkers at transcript and metabolite/lipid level. To this end, blood RNA for sequencing was collected at two time points and blood plasma for mass-spec was collected at three time points (for experimental overview, see Fig. 1a). During the testing period, the MJD84.2 mice had a significantly lower body weight compared to control mice (Fig. 1b). Assessment of an ataxic phenotype using the beamwalk balance tests at 5 time points revealed only one significant difference. This difference was a faster performance of SCA3 mice on the balance beam at 12 months of age (Fig. 1c), likely attributable to the lower bodyweight. The motor and balance performance of the SCA3 mice was identical to the wild-type mice at all other time points tested.

### Individual brain regions are differently affected by mutant ataxin-3

**Fig. 2:**
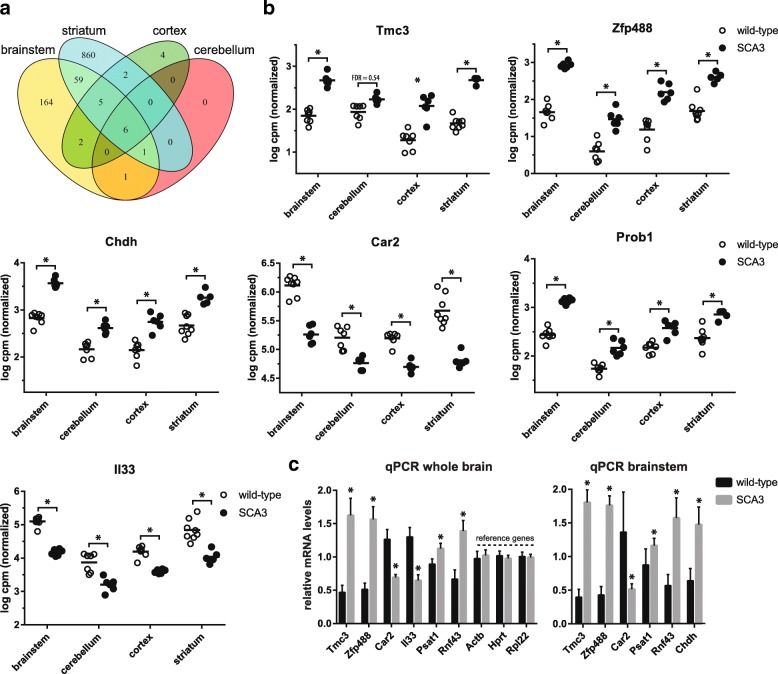
RNA sequencing results for SCA3 mouse brain. **a** Venn diagram depicting overlap of significantly altered genes (FDR < 0.05) from RNA sequencing analysis between SCA3 and wild-type mice per brain region. Six genes were common to all four regions. **b** Plots of the 6 most significantly altered genes in SCA3 mouse brain (combined regions). Expression values of genes are depicted separately for the 4 tested brain regions. * = FDR < 0.01 **c** qPCR validation on equimolar cDNA from the 4 brain regions, as well as separately in brainstem confirms significant gene expression changes. Based on 7 wild-type vs 6 SCA3 mice at 17.5 months of age. * = FDR < 0.01. *Actb, Hprt* and *Rpl22* were used as reference genes

**Fig. 3:**
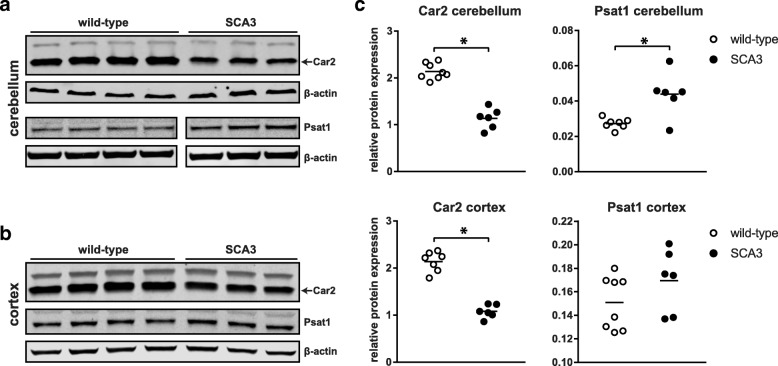
Protein validation of RNA sequencing results in SCA3 mouse brain. Western blot analysis of mouse brain lysates from cerebellum (**a**) and cortex (**b**) probed for Car2 and Psat1 protein. Depicted are results of 4 wild-type and 3 SCA3 mice. **c** Quantification of band intensity reveals significant downregulation of Car2 protein in cerebellum and cortex of SCA3 mice, and significant upregulation of Psat1 in cerebellum. Protein expression was corrected per lane for β-actin levels. Based on 8 wild-type vs 6 SCA3 mice. * = *p*-value < 0.05 with student’s *t*-test

To establish differential gene expression changes between wild-type and SCA3 mice, RNA sequencing of brain and blood tissue was performed (Table 1). After exclusion of RNA samples with low concentration (< 200 ng), a total of 53 samples were successfully sequenced. The average number of reads per sample was 84 million (SD ± 18 million) and on average 66% of sequencing reads were aligned to exons of known genes (Additional file 2: Figure S1). Genes with average expression below 4 CPM were excluded, resulting in a total of 12,372 genes to be included for differential expression analysis. The brain RNA sequencing data can be accessed at GEO repository GSE107958. PCA plots showed good separation of samples based on brain region (Additional file 3: Figure S2) and using an FDR of < 0.05, a total of 585 genes were found significantly altered in the SCA3 brain regions combined analysis. The top 25 genes from the analysis of brain regions combined are listed in Table 2, with corresponding log2 fold change per brain region. When examining each brain region individually, the extent of differential gene expression in SCA3 mice differed greatly per brain region (Fig. 2a), with 238 genes differentially expressed in brainstem, 8 in cerebellum, 19 in cortex and 933 in striatum (FDR < 0.05) compared to wild-type mice. This observation is consistent with smaller fold-changes observed for most genes in cerebellum and cortex. Of the differentially expressed genes, 6 (*Rnf43, Zfp488, Car2, Chdh, Prob1, Il33*) were consistently significantly altered in all 4 brain regions (Fig. 2b). For each brain region that we analysed, we ranked the genes based on *p*-value, and the majority of the genes in these 4 lists were unique to that particular brain region, thus revealing tissue specific gene expression patterns. For validation we selected 6 genes from the top 25 significant genes of the brain region combined analysis, based on FDR, fold change and expression level. Through qPCR on the same samples as used for RNA sequencing, we validated the significant change in expression level for all 6 genes (Fig. 2c). Finally, differential expression was confirmed at the protein level for carbonic anhydrase 2 (*Car2*) and phosphoserine aminotransferase 1 (*Psat1*), as these proteins were predicted to be differentially expressed in all 4 brain regions. Cortex and cerebellum of the SCA3 mice was available for validation of protein levels, and both brain regions showed a similar direction of protein change as was found on mRNA level and reached significance for *Car2* in both brain regions and for *Psat1* in cerebellum (Fig. 3 and Additional file 4: Figure S3).

**Table 2:** Top 25 differentially expressed genes in SCA3 mice brains (regions combined)

Gene symbol	Name	FDR	Brainstem log2 fold change	Cerebellum log2 fold change	Striatum log2 fold change	Cortex log2 fold change	Protein function (GO term mol function or biological process)
*Tmc3*	transmembrane channel-like gene family 3	1.30E-61	1.16^a^	0.42	1.47^a^	1.16^a^	ion transport
*Zfp488*	zinc finger protein 488	1.05E-56	1.79^a^	1.25^a^	1.29^a^	1.45^a^	transcription, oligodendrocyte specific
*Car2*	carbonic anhydrase 2	3.63E-44	−1.26^a^	−0.64^a^	− 1.26^a^	−0.72^a^	carbonate dehydratase activity
*Chdh*	choline dehydrogenase	3.26E-40	1.04^a^	0.66^a^	0.85^a^	0.87^a^	choline dehydrogenase activity
*Prob1*	proline rich basic protein 1	9.30E-38	1^a^	0.62^a^	0.68^a^	0.59^a^	unknown
*Il33*	interleukin 33	9.98E-35	−1.3^a^	−0.98^a^	−1.2^a^	−0.87^a^	cytokine activity
*Fbxw15*	F-box and WD-40 domain protein 15	5.97E-27	−1.8^a^	−0.84	−1.44^a^	−0.79	unknown
*Rnf43*	ring finger protein 43	5.64E-21	1.06^a^	0.74^a^	0.65^a^	0.88^a^	ubiquitin-protein transferase activity
*Polr2a*	RNA polymerase II subunit A	2.00E-20	0.74^a^	0.16	0.47^a^	0.35	DNA-directed RNA polymerase activity
*Ppl*	periplakin	1.50E-19	2.14^a^	0.73	0.9^a^	0.62	cadherin binding involved in cell-cell adhesion
*Arsb*	arylsulfatase B	2.48E-16	0.53^a^	0.2	0.32^a^	0.18	sulphate hydrolysis
*Kcnk13*	potassium two pore domain channel subfamily k member 13	3.04E-16	−0.97^a^	−0.73^a^	−0.81^a^	−0.44	voltage-gated ion channel
*Chil1*	chitinase-3-like protein 1	7.24E-16	−0.75^a^	−0.28	− 0.69^a^	−0.56^a^	carbohydrate metabolic process
*Serpinb1a*	serpin Family B Member 1	1.16E-15	−1.34^a^	−0.86	−1.21^a^	−0.81	negative regulation of endopeptidase activity
*Tspan2*	tetraspanin 2	5.27E-15	−0.87^a^	−0.3	− 0.99^a^	−0.41	astrocyte and microglia development
*Hist1h2be*	Histone H2B type 1-C/E/G	2.39E-14	0.78^a^	0.18	0.77^a^	0.66^a^	antibacterial humoral response
*Acot1*	Acyl-coenzyme A thioesterase 1	1.90E-13	0.74^a^	0.35	0.28	0.29	acyl-CoA metabolic process
*Erbb2ip*	erbin	1.86E-12	−0.89^a^	−0.44	−0.54^a^	− 0.30	cellular response to tumor necrosis factor
*Glul*	Glutamine synthetase	1.12E-11	−0.63^a^	−0.25	− 0.45^a^	−0.21	glutamine biosynthetic process
*Cbs*	Cystathionine beta-synthase	1.68E-11	−0.41^a^	−0.06	− 0.40^a^	−0.14	catalyzes first step of the transsulfuration pathway
*Qdpr*	Dihydropteridine reductase	2.10E-11	−0.64^a^	−0.39	− 0.70^a^	−0.36	6,7-dihydropteridine reductase activity
*Sox8*	Transcription factor SOX-8	6.57E-11	0.58^a^	0.37	0.42^a^	0.38	enteric nervous system development
*Psat1*	Phosphoserine aminotransferase	6.63E-11	0.48^a^	0.39	0.40^a^	0.46^a^	L-serine biosynthetic process
*Enpp6*	Ectonucleotide pyrophosphatase/phosphodiesterase family member 6	2.01E-10	0.20^a^	0.58	0.76^a^	1.25^a^	choline metabolic process
*Ttyh1*	Protein tweety homolog 1	1.37E-09	−0.44^a^	−0.12	−0.04	−0.14	chloride transport

Noted with ^a^are genes that are also differentially expressed in individual brain regions

### Cellular signalling pathways are altered in SCA3 mouse brain

To establish gene expression changes in SCA3 mice at the gene function level, the Euretos knowledge platform and Ingenuity pathway analysis (IPA) tools were used to assess pathway enrichment. Both tools showed good overlap in the top significant pathways for brain region combined analysis. The top pathways associated with the 585 differentially expressed genes in SCA3 mouse brain (4 regions combined) are listed in Table 3. The top pathways are sorted on ingenuity p-value, the complete list of pathway analysis can be found in (Additional file 5: Canonical pathways ingenuity). The combined region pathways signify alterations in pathways which are most consistent for the 4 brain regions, though effect size can differ per individual region. From this combined analysis, cellular signalling pathways were the most significantly enriched pathways, namely: α-adrenergic, CREB and protein kinase A (PKA) signalling, which are all predicted to be downregulated. CREB proteins can be activated by phosphorylation by kinases, including PKA [31], and can thus be involved in the same signalling cascade. Indeed, both CREB and PKA signalling have been implicated in Huntington disease [32, 33] and other neurodegenerative disorders [34], and CREB signalling is known to be required for long-term synaptic plasticity and axonal outgrowth [35], which was also found as one of the most significantly altered pathways. Similar to Huntington disease, sterol regulatory element binding proteins (SREBPs) and cholesterol biosynthesis [36, 37] were also among the top significantly altered pathways in the current SCA3 study. Finally, a total of 24 significantly altered genes were associated with the cellular process of myelination (go:0042552), suggesting a defect in myelin homeostasis in SCA3 brain as was also reported for Huntington disease [38].

**Table 3:** Top overrepresented pathways for genes differentially expressed in SCA3 mouse brain

Pathway	Number of genes	p-value	Pathway database
Brain regions combined analysis (585 genes)			
α-adrenergic signalling	11	1.23E-05	IPA
CREB signalling in neurons	25	1.95E-05	IPA
Protein kinase A signalling	25	2.57E-05	IPA
Axon guidance	24	3.63E-05	IPA + Euretos
Transmission across chemical synapses	13	5.50E-05	IPA + Euretos
Superpathway of cholesterol biosynthesis (srebp)	6	6.03E-05	IPA + Euretos
Myelination (cellular process)	24	8.02E-06	IPA + Euretos
Brainstem (195 genes)			
pi-3 k cascade	6	1.20E-04	IPA + Euretos
amino acid metabolism	9	1.31E-04	Euretos
Superpathway of Cholesterol Biosynthesis	5	1.74E-04	IPA + Euretos
Striatum (824 genes)			
axon guidance	38	2.19E-07	IPA + Euretos
neurotransmitter receptor binding and downstream transmission in the postsynaptic cell	19	9.72E-06	Euretos
synaptic transmission/long term potentiation	23	3.02E-05	IPA + Euretos

Overrepresented pathways based on Ingenuity (IPA) and Euretos pathway analyses. Where applicable, Ingenuity obtained p-values are preferentially reported. The three top pathways in brainstem were also significantly altered in striatum

**Fig. 4:**
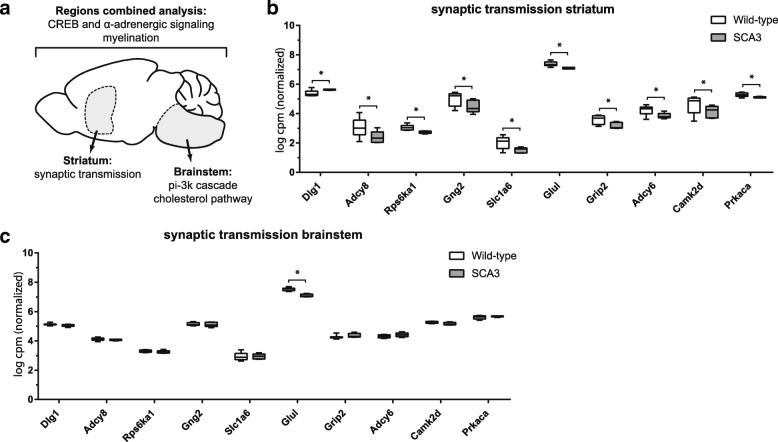
Affected pathways in SCA3 mouse brain. **a** Brainstem and striatum present with different top affected pathways based on gene expression analysis. Expression of synaptic transmission associated genes in striatum (**b**) and brainstem (**c**) of wild-type and SCA3 mice confirm that the transcriptional changes in this pathway are specific to striatum. Obtained from RNA sequencing of 8 wild-type and 6 SCA3 mice. Depicted are 10 out the 23 differentially expressed genes within synaptic transmission pathway

Since ataxin-3 is ubiquitously expressed in brain, and in SCA3 patients there is no clear correlation between the affected brain regions and level of ataxin-3 expression [39], region specific pathological mechanisms are likely at play. Indeed, different pathways were observed when performing brain region combined analysis compared to brainstem and striatum individually (Table 3 and Fig. 4a). In striatum, the predominant effects were observed in axon guidance and synaptic transmission pathways (Fig. 4b) in addition to neurotransmitter receptor induced postsynaptic events. These pathways were however not apparently affected in brainstem (Fig. 4c). Of note, the affected neurotransmitter receptor pathway is most likely glutamate dependent based on involved genes (*Grind2d* and *Grik1*). Transcriptional analysis of SCA1 [40, 41] as well as SCA7 [42] mouse models have previously established a potential involvement of glutamate signalling, suggesting that this may be a signalling pathway that is more broadly affected in the polyQ cerebellar ataxias. Brainstem showed the most significant alterations in amino acid metabolism, cholesterol biosynthesis and the pi-3 k cascade, though these pathways were also significantly altered in striatum. Due to the small number of differentially expressed genes, pathway analysis was not possible for cerebellum and cortex.

### Differential gene expression in blood

**Fig. 5:**
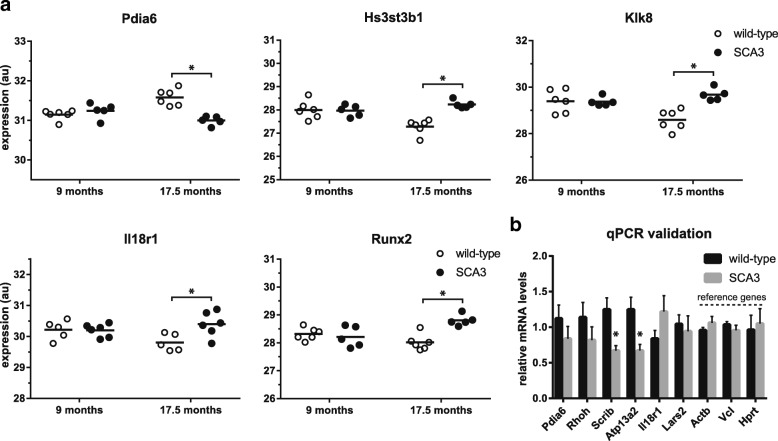
top 5 differentially expressed genes in blood of SCA3 mice. At 17.5 months 142 genes were differentially expressed (FDR < 0.05). **a** Normalized expression of top 5 differentially expressed genes at 17.5 months of age in blood of wild-type and SCA3 mice as detected by RNA sequencing. **b** qPCR validation of blood RNA confirms significant gene expression changes for *Scrib* and *Atp13a2*. Based on 8 wild-type vs 6 SCA3 mice at 17.5 months of age. * = FDR < 0.05. *Actb, Vcl* and *Hprt* (right columns) were used as reference genes

Blood samples were collected at 9 and 17.5 months of age, RNA was isolated and sequenced after depletion of globin transcripts. Average number of reads was 57.5 million (SD ± 10.7 million), and on average 53% were aligned to known genes (Additional file 6: Figure S4A). The blood RNA sequencing data can be found under GEO accession GSE108069. A total of 9800 genes were used for gene expression analysis. Globin transcripts were successfully reduced (Additional file 6: Figure S4B), and were < 4 CPM. However, both average GC percentage and 5′-3′ bias were significantly lower in the samples from SCA3 mice (Additional file 6: Figure S4C and D). The GC content can have a confounding effect on differential gene expression in RNA sequencing analysis, because it may arise during PCR amplification before sequencing, and it is difficult to separate from a true signal [43]. For this reason, GC-content correction was performed prior to analysis [24]. At 9 months of age, only *Uba52* was significantly downregulated in blood of SCA3 mice, while at 17.5 months of age a total of 142 genes were found differentially expressed compared to wild-type mice. The top 10 differentially expressed genes at 17.5 months are listed in Table 4 and corresponding plots of the top 5 genes are shown in (Fig. 5a). Of the significantly altered genes in SCA3 mouse blood, Tnfsf14 (Tumor Necrosis Factor (Ligand) Superfamily, Member 14) has previously been reported to be upregulated in blood of SCA3 patients [44]. Tnfsf14 showed a log fold change of 0.8 in SCA3 mouse blood, with a FDR of 0.048. Through qPCR validation we were able to verify the expression changes in SCA3 mouse blood for protein scribble homolog (*Scrib,* log fold change − 0.4, FDR 0.02) and cation-transporting ATPase 13A2 (*Atp13a2*, log fold change − 0.4, FDR 0.037), and were able to confirm a trend for 4 other genes tested (Fig. 5b). Pathway analysis of the significantly altered genes revealed an effect on respiratory electron transport and mitochondria associated genes.

**Table 4:** Top 10 differentially expressed genes in SCA3 mouse blood at 17.5 months old

Gene symbol	Name	FDR	Log fold change	Protein function (GO term mol. function or biological process)
*Pdia6*	protein disulfide isomerase associated 6	0.002	−0.6	apoptotic cell clearance
*Hs3st3b1*	heparan sulfate (glucosamine) 3-O-sulfotransferase 3B1	0.002	0.9	glycosaminoglycan biosynthetic process
*Klk8*	kallikrein related-peptidase 8	0.004	1.0	endopeptidase activity
*Il18r1*	interleukin 18 receptor 1	0.007	0.7	interleukin-18-mediated signaling pathway
*Runx2*	runt related transcription factor 2	0.007	0.8	ATP binding
*Reck*	reversion-inducing-cysteine-rich protein with kazal motifs	0.007	1.1	endopeptidase inhibitor activity
*Tob1*	transducer of ErbB-2.1	0.007	0.8	receptor tyrosine kinase binding
*Phf13*	PHD finger protein 13	0.007	0.6	chromatin binding
*Rhoh*	ras homolog family member H	0.007	−0.5	mast cell activation
*Smad7*	Mothers Against Decapentaplegic Homolog 7	0.010	0.7	activin binding

### Metabolic and lipid changes in blood of SCA3 mice

Plasma samples from 4 wild-type and 4 transgenic mice were collected at 4, 12 and 16 months of age and used for LC-MS detection of metabolites (Profilomics, Gif-sur-Yvette, France). A total of 195 variables were detected in both ionization modes, where 114 could be matched at a level 1 annotation (retention time, relative isotopic ratio and MS/MS spectra) and 81 with a level 2 annotation (no MS/MS data) to an in-house database of metabolites. Combining positive and negative ion modes led to detection of 148 unique metabolites. The corresponding chemical classes of the detected metabolites are depicted in (Additional file 7: Figure S5).

**Fig. 6:**
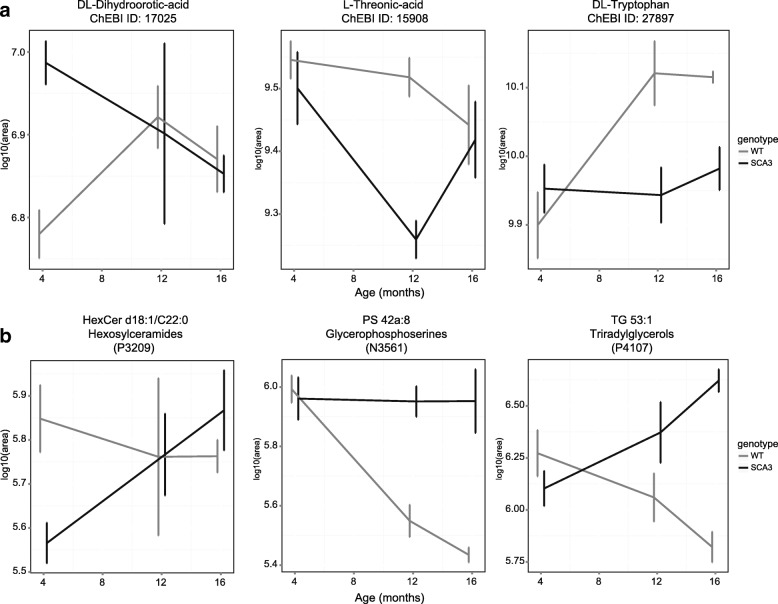
Significantly altered metabolites at 4, 12 and 16 months of age in the MJD84.2 mouse model. **a** Levels of the 3 most significantly altered metabolites over time. **b** Levels of 3 most significantly altered lipids over time. Listed profilomic ID can be found in (Additional file 9 and 11). Based on 4 wild-type vs 4 SCA3 mice. Depicted is mean log areas ±SD per time point

Alterations in metabolite levels were assessed between wild-type and SCA3 mice at individual time points using the Welch’s unequal variances *t*-test procedure by comparing the area under the curve (AUC) using log10 areas. Due to the low sample number, there was no correction for multiple testing and nominal *p*-values are reported. A total of 32 metabolites were found to be significantly different (*p* < 0.05) between SCA3 and wild-type mice. The 10 most significantly altered metabolites, irrespective of testing time point, are listed in Table 5. At 4 months of age, DL-Dihydroorotic-acid was most significantly altered, whilst L-Threonic-acid was most significantly altered at 12 months of age, and DL-tryptophan at 16 months Fig. 6a).

**Table 5:** Top altered blood metabolites in SCA3 mice at 3 time points

Compound	ChEBI ID	Fold change	*p*-value	Altered at Time points	Associated pathway
4 months					
DL-Dihydroorotic-acid	17025	1.61 ± 0.19	0.002	4 months	Pyrimidine Metabolism
N-a-acetyl-L-arginine	40521	1.95 ± 0.39	0.003	4 months	NA
3-hydroxydecanoic-acid / 10-hydroxydecanoic-acid	17409	1.51 ± 0.21	0.005	4 months	Fatty Acid
12 months					
L-Threonic-acid	15908	0.55 ± 0.08	0.001	12 months	Ascorbate and aldarate metabolism
2-Aminoisobutyric-acid / Aminobutyric-acid	27971	2.58 ± 0.69	0.002	12 months	NA
Asparagine	17196	0.68 ± 0.08	0.003	12 months	Ammonia Recycling / Aspartate Metabolism / Transcription/Translation
16 months					
Methylhistamine	29009	0.87 ± 0.06	0.019	16 months	Histidine Metabolism
DL-Tryptophan	27897	0.74 ± 0.11	0.020	12 and 16 months	NA
Threonine / D-allo-Threonine	16857	0.77 ± 0.09	0.038	16 months	Glycine and Serine Metabolism / Threonine and 2-Oxobutanoate Degradation / Transcription/Translation

Nominal p-values reported, associated pathway obtained from Profilomics database

To assess alterations of the metabolome in SCA3 mice over time, a PCA was performed (Additional file 8: Figure S6). Age was weakly but significantly correlated with the first principal component (PC), which explains 57% of variance (ρ = − 0.586, *p* < 0.05). Genotype also weakly but significantly correlated with PC3, explaining 7% of variance (Additional file 8: Figure S6B) (ρ = − 0.463, *p* < 0.05), indicating that the effect of mutant ataxin-3 expression in the mice did not induce a strong effect on blood metabolite levels. When comparing SCA3 to wild-type mice at 4, 12 and 16 months of age, the number of significantly altered metabolites in blood were 14, 20 and 4 respectively. From these metabolites, only DL-Tryptophan was altered at two of the time points, whilst the other metabolites were only found to be altered at a single time point. The full list of measured metabolites and comparisons between genotypes can be found in (Additional file 9: Blood metabolites).

On the same plasma samples, lipid levels were also examined. A total of 491 unique lipids were identified, divided over 26 classes (Additional file 7: Figure S5). To have an overview of the dataset, areas of all unique lipids from the same lipid class were summed. Differences in levels of the individual lipids and of the lipid classes at 4, 12 and 16 months were assessed using the Welch’s unequal variance t-test without multiple testing correction (Additional file 10: Table S2). Using this method, at 4 months of age no lipid classes were found significantly different in plasma between SCA3 and wild-type mice. At 12 months of age, glycerophosphoserine and sulfatides were decreased significantly in the SCA3 mouse. At 16 months of age, di- and triacylglycerols and ceramides were significantly increased in plasma of SCA3 mice compared to wild-type. Both diacylglycerols and ceramides have been linked to the oxidative stress and stress signalling pathways [45, 46]. In contrast, glycerophosphoserine, lyso-phosphoinositols and sulfatide were found to be decreased in the SCA3 mice over time. Interestingly, 3 of the 4 NeuGC-GM2 gangliosides were found significantly altered at 16 months. The full list of measured lipids can be found in (Additional file 11: Blood lipids). The most significantly altered individual lipids are shown in (Fig. 5b). Due to their association with disease progression, ceramides, sulfatides, glycerophosphoserine and triradylglycerol may be of potential interest as biomarkers of disease progression in these mice.

## Discussion

Here, we determined gene expression as well as metabolite and lipid changes in the SCA3 MJD84.2 mouse model [16]. Transcriptional deregulation is a known pathogenic process in SCA3 [8], but so far few studies have been performed to establish which transcriptional changes occur and how these are involved in the molecular pathogenicity in SCA3. Furthermore, there is currently a requirement for reliable (pre)clinical biomarkers capable of tracking disease progression in SCA3.

### Multi-omic biomarker identification in blood of SCA3 mice

Both metabolites [47] and gene transcripts [48] may serve as biomarkers to track neurodegenerative disease progression in blood. Sequencing of whole blood RNA revealed lower levels of *Uba52* at 9 months of age, whereas 142 genes were differentially expressed at 17.5 months of age in the SCA3 mice. A total of 10 genes have been reported as transcript biomarkers in blood of SCA3 patients [44]. Of these 10 genes, only upregulation of Tumor Necrosis Factor Superfamily Member 14 (*Tnfsf14)* was also observed significantly upregulated in our dataset of the SCA3 mice. Despite the modest overlap, this observation does solidify *Tnfsf14* as a potential blood biomarker for SCA3. Pathway analysis of the 142 altered genes in our blood dataset suggested affected respiratory electron transport pathways, in line with mitochondrial abnormalities and increased oxidative damage observed in peripheral blood of Huntington patients [49] and mitochondrial DNA damage previously reported in blood and brain of SCA3 mice [50]. Interestingly, whole blood RNA sequencing of SCA2 patients also suggested affected mitochondrial function [51], suggesting a potential commonality between the different polyQ disorders.

Metabolite analysis of blood revealed a range of altered metabolites in SCA3 mouse blood at all three time points tested. However, due to the small sample size used, the results must be interpreted with caution and the most relevant alterations in metabolites are those that are represented at multiple time points and show increasing fold change over time. In this regard, DL-Tryptophan (CHEBI: 27897) was identified as the most promising biomarker. DL-tryptophan levels were found to be altered at both 12 and 16 months of age, with lower levels in SCA3 mice (fold change 0.7 +/− 0.11). Interestingly, blood tryptophan levels have been correlated with disease progression in blood of Huntington disease patients, with affected patients also showing lower levels [52, 53]. Indeed, tryptophan and its degradation products have been proposed as pathogenic factors in Huntington brain, with the tryptophan metabolite quinolinate reported to be elevated in Huntington disease brain, due to increased 3-hydroxyanthranilate oxygenase activity [54]. To our knowledge, tryptophan levels in blood of SCA3 patients have not been assessed yet, and would thus be a good starting point to establish a biomarker indicative of disease progression.

Lipidomic analyses revealed that at 16 months of age the di- and triglycerides and ceramides (CHEBI: 85812 and 85777) levels were increased considerably in the SCA3 mice (Additional file 9: Blood metabolites). Interestingly, increased triglycerides levels have been detected in blood of SCA3 patients [55], but ceramides have not yet been assessed in a clinical setting. In a mouse model for Huntington disease, increased diacylglycerol kinase (DGK) activity has been observed, and a protective effect of DGK inhibition was suggested [56]. In line with the blood transcriptional changes, ceramides have been frequently reported in relation with neurodegenerative disorders, especially in the context of oxidative stress, inflammation and apoptosis [57–59]. For instance, in spinal cord tissue from amyotrophic lateral sclerosis spinal cord patients, increased levels of ceramides were detected and preceded the clinical phenotype in a mouse model [60]. The proposed mechanism is that the mutant protein leads to increased oxidative stress, thereby altering the sphingolipid metabolism to produce more ceramides and cholesterol esters, in turn sensitising motor neurons susceptible to excitotoxicity and oxidative stress, culminating in cell death [60]. A comparison between ceramides in blood and CNS tissue of the SCA3 mouse in future experiments may thus be useful to establish ceramides as a potential biomarker.

### CREB and α-adrenergic signalling pathway transcripts are most consistently altered throughout the SCA3 mouse brain

A combined brain region differential gene expression analysis was performed in order to prioritise the most robust and consistent transcriptional alterations across all brain regions. In this manner, CREB and α-adrenergic signalling pathways were determined as most strongly affected in the SCA3 mouse brain. α-Adrenergic signalling has not yet been extensively investigated for SCA3, and further validation in other mouse models and patient brain material should thus be performed to more reliably establish this finding. However, adenosine homeostasis is reportedly changed in Huntington [61], suggestive of potential parallels between the two polyQ disorders. Additionally, an adenosine A2A receptor agonist, though pleiotropic, was shown to have beneficial effects on neurodegeneration and transcriptional dysregulation in a SCA3 transgenic mouse [62].

Downregulation of CREB signalling was the second most affected pathway based on the RNA sequencing of brain tissue in the SCA3 mice. This finding is in good agreement with previous studies where ataxin-3 was found to interact with CREB-binding protein, and inhibits transcription by this coactivator [63].This inhibition likely takes place through sequestration of CREB-binding protein by the polyglutamine, as evidenced in the polyQ disease spinal and bulbar muscular atrophy (SBMA) [64]. Furthermore, an expanded polyglutamine stretch is also known to supress phosphorylation of CREB through binding of the coactivator TAFII130, interfering with CREB-dependent transcription and subsequently contributing to polyQ pathogenicity [65]. Also, CREB deficiency enhances polyQ induced lethality in *Drosophila,* which can be partly rescued by increased CREB expression [66]. As CBP regulates CREB [67] and SREBP transcriptional activity [68], these results suggest that loss of CBP function underlies at least part of the transcriptional dysregulation in the SCA3 brain, similar to what has been suggested for Huntington disease [69]. Consistent with the synaptic transmission related gene expression changes we observed in striatum of the SCA3 mice, CREB signalling is known to be required for long-term synaptic plasticity and axonal outgrowth [35]. Together, these findings suggest that CREB dependent transcription is indeed inhibited due to presence of expanded polyQ protein, and that the resulting transcriptional dysregulation contributes to the pathogenic mechanisms in SCA3 [34].

The relation between cellular dysregulation, neuronal loss, cerebellar dysfunction and the onset of motor/coordination symptoms in SCA3 is not yet elucidated. Other reports using the MJD84.2 mouse found that changes in Purkinje cell firing are an early disease manifestation that occur prior to observable neurodegeneration, but coincide with behavioural deficits of the mice [70]. Costa et al. also reported onset of behavioural deficits in the homozygous MJD84.2 mice, with unaltered Purkinje cell counts at the same time point [71]. The 75 week time point used for transcriptional analysis in this study corresponds to the early and minor loss of Purkinje cells in the MJD84.2 mouse model reported by others [70], but there were no behavioural deficits in the current study. Other molecular hallmarks of SCA3 are however conclusively present in these mice at this time point, including increased ataxin-3 nuclear localisation and insolubility [71–73]*,* which is considered an early stage of aberrant protein aggregation, deranged calcium signalling [72] and the increased excitability in Purkinje cells [70].

### Mutant ataxin-3 affects synaptic transmission pathways more strongly in striatum

From the combined brain region transcriptional analysis, CREB and α-adrenergic signalling were found most strongly affected. However, it was clear that the contribution of each individual brain region to this list was not equal. We observed larger fold changes and more differentially expressed genes in striatum and brainstem than observed in cortex and cerebellum. As we and others have repeatedly shown similar expression of the mutant ataxin-3 transgene in the MJD84.2 mouse model in the brain regions tested here [16, 73, 74], it is unlikely that variations in expression levels can explain these differences. Since previous studies suggest that cellular *ATXN3* transcript and protein levels do not correlate well with neuronal degeneration in SCA3 [39, 75], these findings are indicative of differential effects of mutant ataxin-3 in each brain region. One of the more surprising findings in our dataset was the fact that the synaptic transmission pathways were more strongly affected in striatum compared to brainstem and cerebellum. Pathway analysis of the transcriptome in the brainstem showed that the pi-3 k cascade and cholesterol biosynthesis pathways were most significantly altered in this brain region of the SCA3 mouse. It is not clear why different pathways are affected in brainstem compared to striatum in the SCA3 mouse. However, in a previous study we did note the strongest nuclear localisation of mutant ataxin-3 in the substantia nigra [73]. In SCA3 patients a marked reduction in dopamine transport was found in striatum [76]. Given that the dopaminergic innervation of striatum originates from substantia nigra [77, 78], pathogenic nuclear localisation of mutant ataxin-3 may interfere with this dopaminergic signalling. Indeed, in light of the requirement of CREB for dopamine dependent gene expression in the striatum [79], the observed alteration in CREB signalling in the striatum of the SCA3 mouse may reflect affected dopaminergic signalling from substantia nigra. Nonetheless, in a more severe SCA3 mouse model synaptic transmission and signal transduction pathways were found altered in cerebellum of symptomatic mice [8]. It will thus be interesting to determine whether these synaptic transmission deficiencies in cerebellum correlate with nuclear localisation or aggregation of mutant ataxin-3 and are a requirement for motor phenotype onset. The affected axon guidance pathway in striatum of SCA3 mice was also identified in a transcriptomic study with SCA2 mice, where weighted correlation network analysis of cerebellum found one module associated with axon guidance correlating to disease status [80].

### Emerging role of white matter dysfunction in SCA3

In a recent study, RNAseq profiling was performed on pons of 22 week old MJD84.2 and two knock-in SCA3 models [81]. A total of 38 genes were found differentially expressed in pons of these mouse models. In our study, we were able to identify 32 of these reported genes, and indeed found significant differential expression for 23 of those genes in brainstem of the MJD84.2 mice. This overlap argues for the robustness of both studies, and since we observed altered expression for 11 genes associated with myelination (*Olig1, Olig 2, Ddx54, Fyn, Egfr, Cdkn1c, Pmp22, Klk6, Mal, Tspan2,* and *Aspa*), our findings further solidify white matter changes as a potential disease process in brainstem of SCA3 mice. The top downregulated protein identified in our study, Car2, accumulates on oligodendrocyte processes associated with myelinated axons and it is thought that Car2 may be involved in myelin formation in the central nervous system [82], though no major myelin abnormalities have been observed in Car2 deficient mice [83, 84]. Furthermore, *Zfp488* (zinc finger protein 488) was significantly upregulated in SCA3 mice, and plays a role in the differentiation of neural progenitor cells to mature oligodendrocytes, thereby assisting in remyelination after injury [85]. Together, these gene expression studies warrant further investigation of these white matter related processes in SCA3 pathogenicity.

## Conclusions

Taken together, we report here *Tnfs14* transcript, DL-tryptophan levels and spingolipids ceramides as potential blood biomarkers for SCA3. Mechanistically, we found alterations in transcript levels for CREB and α-adrenergic pathways most consistently affected throughout all brain regions of the MJD84.2 mice. In striatum, synaptic transmission pathways were most strongly affected, whilst brainstem showed largest changes in the pi-3 k cascade.

## Additional files

Additional file 1: **Table S1.** Primers used for qPCR validation of RNA sequencing results. (DOCX 13 kb)

Additional file 2: **Figure S1.** Brain alignment summary. (PDF 116 kb)

Additional file 3: **Figure S2.** Brain PCA. (PDF 141 kb)

Additional file 4: **Figure S3.** Protein validation of RNA sequencing results in SCA3 mouse brain. Western blot analysis of mouse brain lysates from cerebellum (**a**) and cortex (**b**) probed for Car2 and Psat1 proteins. Uncropped blots from those shown in Fig. 3, showing all 8 wild-type and 6 SCA3 mice. (JPG 434 kb)

Additional file 5: Canonical pathways ingenuity. (XLSX 257 kb)

Additional file 6: **Figure S4.** number of reads and quality of blood RNA sequencing. **A** Number of reads obtained for each mouse is depicted per time point. RNA sequencing reads were aligned to mouse reference genome build 10 (GRCm38/mm10) using star aligner. **B** Distribution of reads for blood RNA sequencing indicate that globin reduction was efficient (1st rank gene account for < 10% of reads) and read distribution between samples was comparable. *n* = 22. **C** Median 5′-3′ bias in reads per genotype in blood at 17.5 months of age. SCA3 mice show significantly lower values (*p* < 0.05, Welch 2 sample t-test). **D** Average GC percentage of all reads per genotype in blood at 17.5 months. Significantly lower values are seen in blood of SCA3 mice (p < 0.05, Welch 2 sample t-test) prior to GC-content correction. (PDF 472 kb)

Additional file 7: **Figure S5.** distribution of metabolite classes and lipid families identified from mass-spec analysis of plasma samples. LC-HRMS analysis of plasma samples led to identification of 148 unique metabolites and 491 lipids. (PDF 88 kb)

Additional file 8: **Figure S6.** principal component analysis (PCA) of measured metabolites. Individual barcodes of mice are depicted, plasma was obtained for each mouse at 3 time points **A)** Age is significantly correlated with PC1 (ρ = − 0.586, p < 0.05), hence explaining most of the variation between samples. SCA3 *n* = 4, wild-type (WT) *n* = 4. **B)** The third principal component (PC) is significantly correlated with genotype (ρ = − 0.463, p < 0.05). PC3 and PC4 are shown. (PDF 280 kb)

Additional file 9: Blood metabolites. (XLSX 38 kb)

Additional file 10: **Table S2.** Lipid classes altered at 4, 12 and 16 months in SCA3 mice. Only lipid classes with at least one significantly altered unique lipid at any time are shown. (DOCX 14 kb)

Additional file 11: Blood lipids. (XLSX 82 kb)

## Abbreviations

Actb: β-actin
Atp13a2: Cation-transporting ATPase 13A2
ATXN3: Ataxin-3
Car2: Carbonic anhydrase 2
CBP: CREB binding protein
CHEBI: Chemical entities of biological interest
CQN: Conditional quantile normalization
CREB: cAMP response element binding
DGK: Diacylglycerol kinase
FDR: False discovery rate
Hprt: Hypoxanthine-guanine phosophoribosyltransferase
IPA: Ingenuity Pathway Analysis
MJD: Machado-Joseph disease
PC: Principal component
PCA: Principal component analysis
PKA: Protein kinase A
PolyQ: Polyglutamine
Psat1: Phosphoserine aminotransferase 1
Rpl22: Ribosomal Protein L22
SBMA: Spinal and bulbar muscular atrophy
SCA3: Spinocerebellar ataxia type 3
Scrib: Protein scribble homolog
SREBP: Sterol regulatory element binding proteins
Tnfsf14: Tumor Necrosis Factor (Ligand) Superfamily, Member 14
Vcl: Vinculin
