# Supplementary material for: Transcriptional profiling and biomarker identification reveal tissue specific effects of expanded ataxin-3 in a spinocerebellar ataxia type 3 mouse model
Source: Mol Neurodegener. 2018 Jun 22;13:31. doi: 10.1186/s13024-018-0261-9 (PMC6013885; doi:10.1186/s13024-018-0261-9)
Supplement: Supplementary file 1 — Table S1. Primers used for qPCR validation of RNA sequencing results. (DOCX 13 kb) [file 13024_2018_261_MOESM1_ESM.docx]

**Table S1:** Primers used for qPCR validation of RNA sequencing results

| **Target gene** | **Primer name** | **Application** | **Sequence (5′ to 3′)** |
| --- | --- | --- | --- |
| Atxn3 | hATXN3_int_9_fw_56FAM | repeat sizing | /56-FAM/GTAATCTGTATCAGACTAACTGCTCTTG |
| Atxn3 | hATXN3_ex10_rev | repeat sizing | GATGTGAACTCTGTCCTGATAGGTC |
| *Car2* | mCar2_Qex3_Fw | qPCR | GCAGTGCTGAAAGGAGGACC |
| *Car2* | mCar2_Qex4_Rev | qPCR | CCCATATTTGGTGTTCCAGTGAA |
| *Psat1* | mPsat1_Qex1_Fw | qPCR | AAGCCACCAAGCAAGTGGTTA |
| *Psat1* | mPsat1_Qex2_Rev | qPCR | GATGCCGAGTCCTCTGTAGTC |
| *Il33* | mIl33_Qex2_Fw | qPCR | TCCAACTCCAAGATTTCCCCG |
| *Il33* | mIl33_Qex3_Rev | qPCR | CATGCAGTAGACATGGCAGAA |
| *Tmc3* | mTmc3_Qex4_Fw | qPCR | TCATCCCCTGGGAAATGAGGA |
| *Tmc3* | mTmc3_Qex5_Rev | qPCR | TCGGGAAGGACAACAAAGGC |
| *Zfp488* | mZfp488_Qex2_Fw | qPCR | GGCAGGGATGTTCAAGAAAATGA |
| *Zfp488* | mZfp488_Qex2_Rev | qPCR | CAGTCGAGGCTTGTTCGGT |
| *Rnf43* | mRnf43_Qex7_Fw | qPCR | CCGGGTCATTTCGTGCCTC |
| *Rnf43* | mRnf43_Qex8_Rev | qPCR | CCTGGTTCCTGGTAAGATGGAG |
| *Pdia6* | mPdia6_ex11_fw | qPCR blood | CTTCTCAAGGGGTCTTTCAGTG |
| *Pdia6* | mPdia6_ex12_rev | qPCR blood | GTGATGGTAGGAAAGGAACCAC |
| *Rhoh* | mRhoh_ex3_fw | qPCR blood | TTCACGAGTCATTCGCACAC |
| *Rhoh* | mRhoh_ex4_rev | qPCR blood | AGTAAGGAAACGGCAACCAG |
| *Scrib* | mScrib_ex9_fw | qPCR blood | AGCTGATCCTCACGGAGAAC |
| *Scrib* | mScrib_ex10_rev | qPCR blood | CAGGCGATTGTCTCTCAAAG |
| *Atp13a2* | mAtp13a2_Qex12_Fw | qPCR blood | TGACTCGGACAGGGTTCTG |
| *Atp13a2* | mAtp13a2_Qex13_Rev | qPCR blood | GCCACAAACTTCATGCTGTG |
| *Il18r1* | mIl18r1_Qex4_Fw | qPCR blood | TGAAGAGCTGATCCAGGACAC |
| *Il18r1* | mIl18r1_Qex5_Rev | qPCR blood | TCATCTCCAAACTCGGCATC |
| *Lars2* | mLars2_Qex11_Fw | qPCR blood | ATGGCACAGAGAGACTGAGTG |
| *Lars2* | mLars2_Qex12_Rev | qPCR blood | AGCCAGTCCTTCAGCTTGTTAC |
| *Actb* | mActb_Qex2_Fw | qPCR reference | GGCTGTATTCCCCTCCATCG |
| *Actb* | mActb_Qex3_Rev | qPCR reference | CCAGTTGGTAACAATGCCATGT |
| *Hprt* | m_Hprt_F | qPCR reference | TCCCTGGTTAAGCAGTACAGCC |
| *Hprt* | m_Hprt_R | qPCR reference | CGAGAGGTCCTTTTCACCAGC |
| *Rpl22* | mRpl22_ex3_fw1 | qPCR reference | AGGAGTCGTGACCATCGAAC |
| *Rpl22* | mRpl22_ex3_rev1 | qPCR reference | TTTGGAGAAAGGCACCTCTG |
| *Vcl* | mVcl_Qex1_Fw | qPCR reference | TGGACGGCAAAGCCATTCC |
| *Vcl* | mVcl_Qex2_Rev | qPCR reference | GCTGGTGGCATATCTCTCTTCAG |
