# Supplementary figures and images for: Transcriptional profiling and biomarker identification reveal tissue specific effects of expanded ataxin-3 in a spinocerebellar ataxia type 3 mouse model
Source: Mol Neurodegener. 2018 Jun 22;13:31. doi: 10.1186/s13024-018-0261-9 (PMC6013885; doi:10.1186/s13024-018-0261-9)

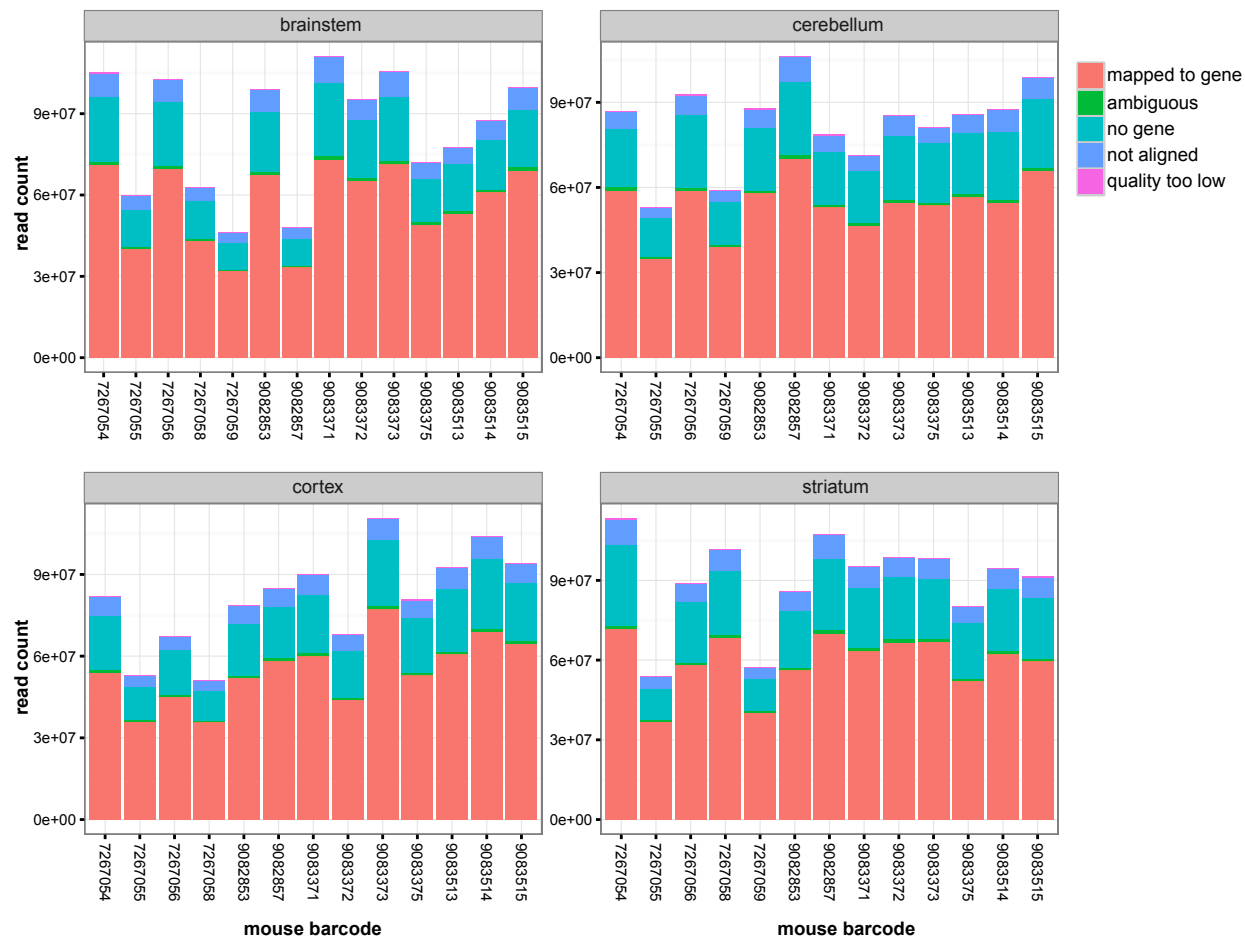

Supplement: Supplementary file 2 — Figure S1. Brain alignment summary. (PDF 116 kb) [file 13024_2018_261_MOESM2_ESM.pdf]

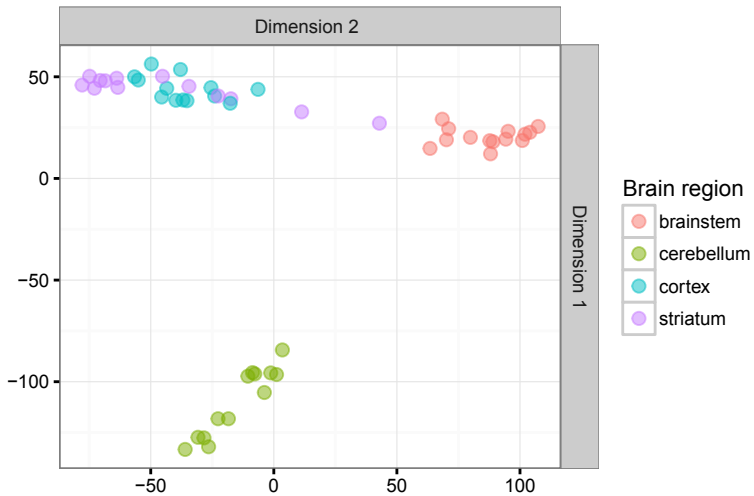

Supplement: Supplementary file 3 — Figure S2. Brain PCA. (PDF 141 kb) [file 13024_2018_261_MOESM3_ESM.pdf]

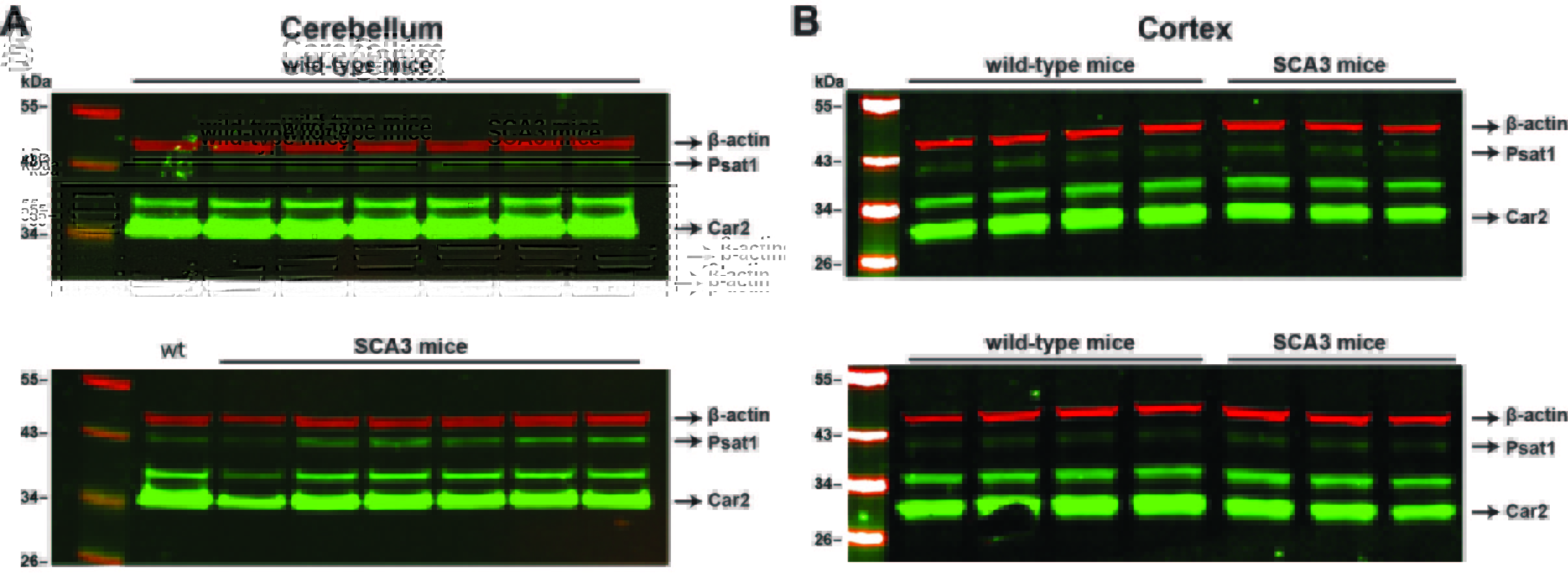

Supplement: Supplementary file 4 — Figure S3. Protein validation of RNA sequencing results in SCA3 mouse brain. Western blot analysis of mouse brain lysates from cerebellum (a) and cortex (b) probed for Car2 and Psat1 proteins. Uncropped blots from those shown in Fig. 3, showing all 8 wild-type and 6 SCA3 mice. (JPG 434 kb) [file 13024_2018_261_MOESM4_ESM.jpg]

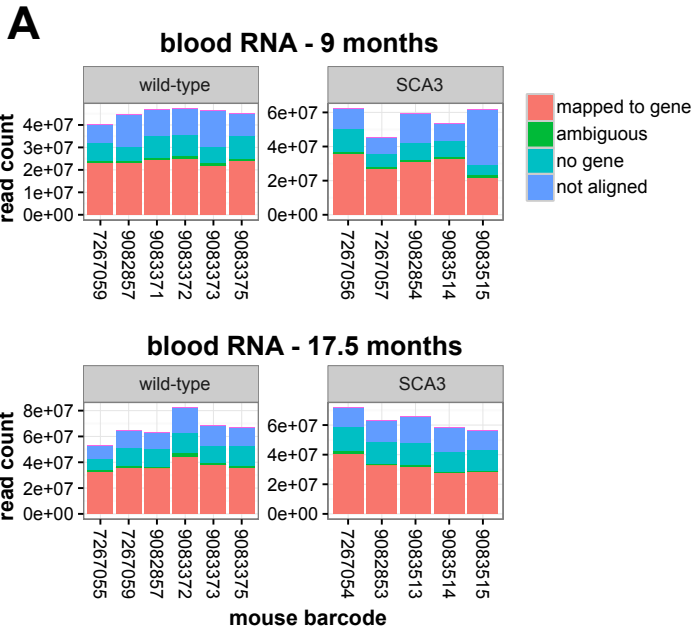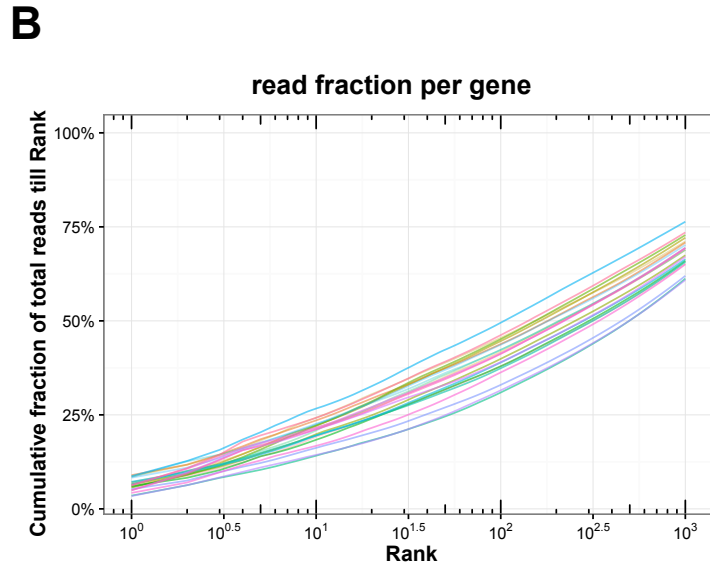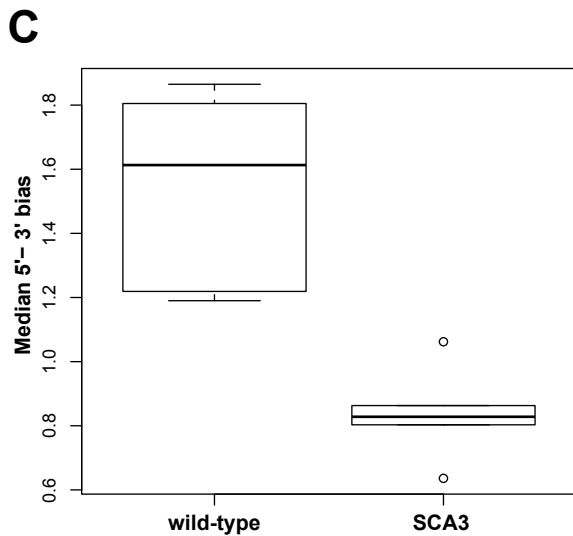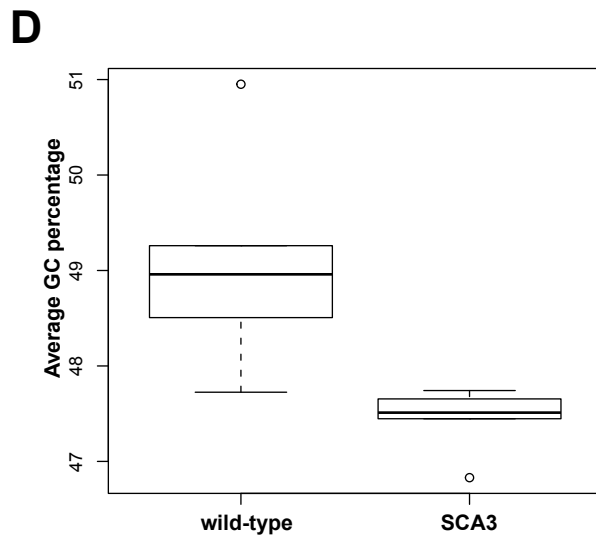

Supplement: Supplementary file 6 — Figure S4. number of reads and quality of blood RNA sequencing. A Number of reads obtained for each mouse is depicted per time point. RNA sequencing reads were aligned to mouse reference genome build 10 (GRCm38/mm10) using star aligner. B Distribution of reads for blood RNA sequencing indicate that globin reduction was efficient (1st rank gene account for < 10% of reads) and read distribution between samples was comparable. n = 22. C Median 5′-3′ bias in reads per genotype in blood at 17.5 months of age. SCA3 mice show significantly lower values (p < 0.05, Welch 2 sample t-test). D Average GC percentage of all reads per genotype in blood at 17.5 months. Significantly lower values are seen in blood of SCA3 mice (p < 0.05, Welch 2 sample t-test) prior to GC-content correction. (PDF 472 kb) [file 13024_2018_261_MOESM6_ESM.pdf]

## Metabolite classes identified

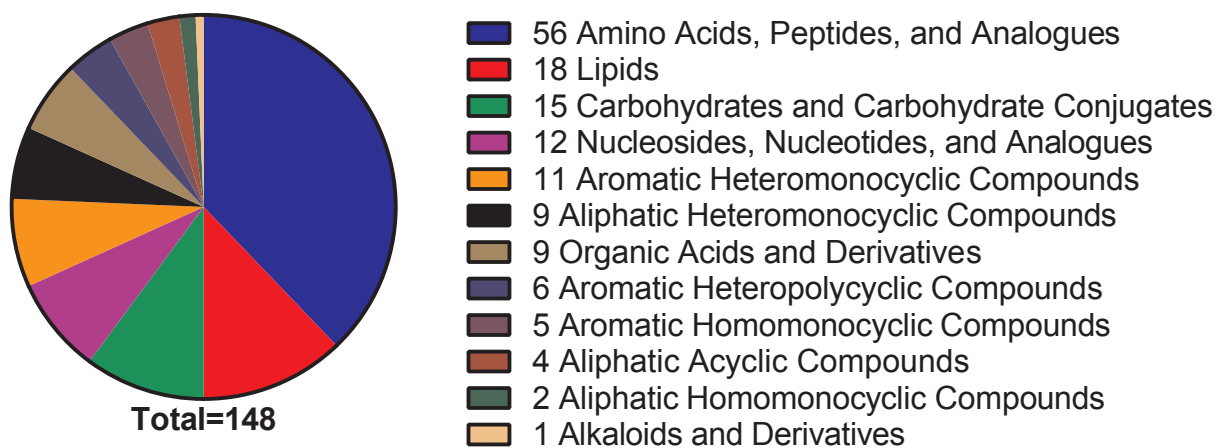

## Lipid families identified

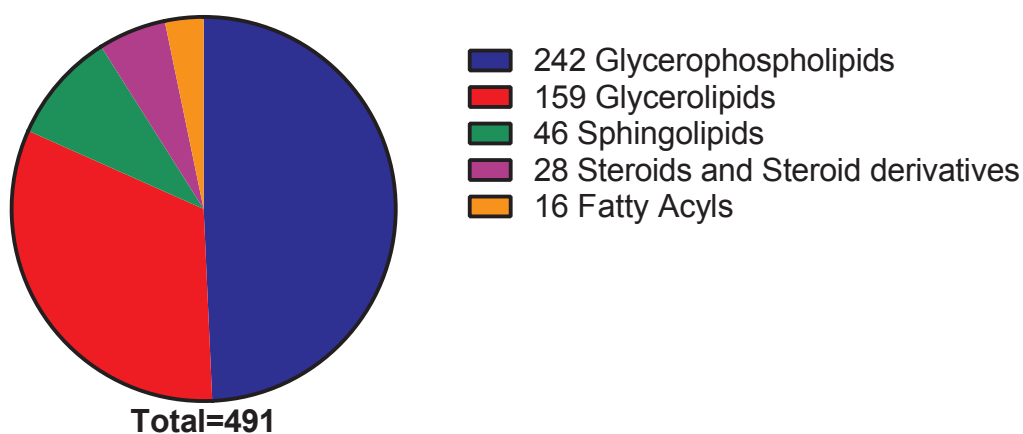

Supplement: Supplementary file 7 — Figure S5. distribution of metabolite classes and lipid families identified from mass-spec analysis of plasma samples. LC-HRMS analysis of plasma samples led to identification of 148 unique metabolites and 491 lipids. (PDF 88 kb) [file 13024_2018_261_MOESM7_ESM.pdf]

**A**

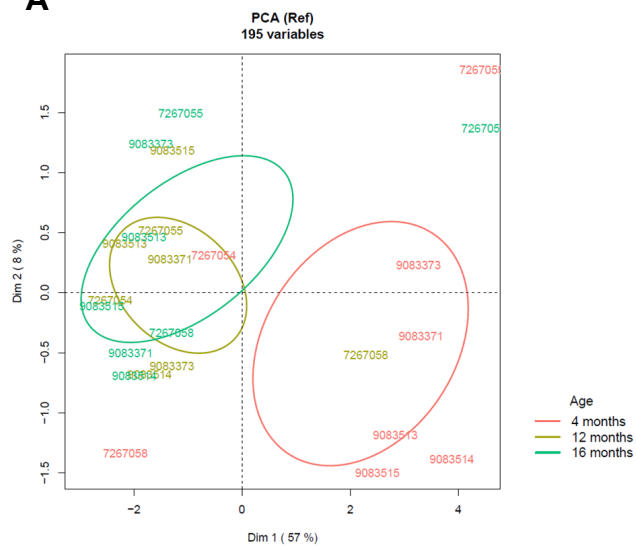

**B**

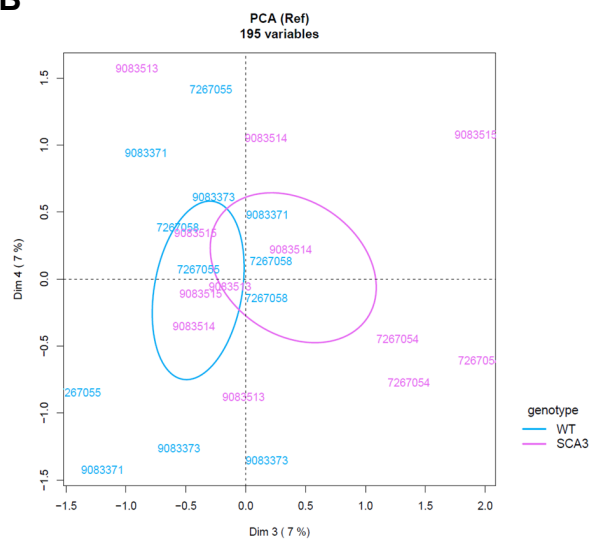

Supplement: Supplementary file 8 — Figure S6. principal component analysis (PCA) of measured metabolites. Individual barcodes of mice are depicted, plasma was obtained for each mouse at 3 time points A) Age is significantly correlated with PC1 (ρ = − 0.586, p < 0.05), hence explaining most of the variation between samples. SCA3 n = 4, wild-type (WT) n = 4. B) The third principal component (PC) is significantly correlated with genotype (ρ = − 0.463, p < 0.05). PC3 and PC4 are shown. (PDF 280 kb) [file 13024_2018_261_MOESM8_ESM.pdf]
