# Supplementary material for: Transcriptional profiling and biomarker identification reveal tissue specific effects of expanded ataxin-3 in a spinocerebellar ataxia type 3 mouse model
Source: Mol Neurodegener. 2018 Jun 22;13:31. doi: 10.1186/s13024-018-0261-9 (PMC6013885; doi:10.1186/s13024-018-0261-9)
Supplement: Supplementary file 10 — Table S2. Lipid classes altered at 4, 12 and 16 months in SCA3 mice. Only lipid classes with at least one significantly altered unique lipid at any time are shown. (DOCX 14 kb) [file 13024_2018_261_MOESM10_ESM.docx]

**Table S2:** Lipid classes altered at 4, 12 and 16 months in SCA3 mice. Only lipid classes with at least one significantly altered unique lipid at any time are shown.

| **Lipid Classes** | **Total number of lipids per class** | **4 months** | | **12 months** | | **16 months** | | **General trend** |
| --- | --- | --- | --- | --- | --- | --- | --- | --- |
|  |  | Number of significant lipids (p<0.05) | p-value* | Number of significant lipids (p<0.05) | p-value* | Number of significant lipids (p<0.05) | p-value* |  |
| Diacylglycerols (DG) | 12 | 0 | ns | 0 | ns | 8 | 0.012 | up |
| Triradylglycerols (TG) | 147 | 0 | ns | 5 | ns | 104 | 0.005 | up |
| Glycerophosphocholines (PC) - acids | 68 | 0 | ns | 10 | ns | 6 | ns |  |
| Glycerophosphoethanolamines (PE) - acids | 27 | 1 | ns | 1 | ns | 4 | ns |  |
| Glycerophosphoinositols (PI) - acyls | 19 | 0 | ns | 4 | ns | 4 | ns |  |
| Glycerophosphoserines (PS) - acids | 10 | 0 | ns | 3 | 0.046 | 4 | 0.006 | down |
| Glycerophosphoserines (PS) - non-acids | 6 | 0 | ns | 1 | ns | 1 | ns |  |
| Lyso-Glycerophosphocholines (LPC) - acids | 29 | 1 | ns | 6 | ns | 2 | ns |  |
| Lyso-Glycerophosphoethanolamines (LPE) - acids | 10 | 0 | ns | 1 | ns | 0 | ns |  |
| Lyso-Glycerophosphoinositols (LPI) - acids | 3 | 0 | ns | 0 | ns | 0 | 0.045 | down |
| Ceramides (Cer) | 3 | 0 | ns | 0 | ns | 2 | 0.039 | up |
| Hexosylceramides (HexCer) | 2 | 1 | ns | 0 | ns | 0 | ns |  |
| Gangliosides - GM2 | 4 | 0 | ns | 0 | ns | 3 | ns |  |
| Sulfoglycosphingolipids (Su) | 5 | 0 | ns | 0 | ns | 1 | ns |  |
| Hydroxylated Sulfoglycosphingolipids (Su-OH) | 3 | 0 | ns | 1 | 0.041 | 3 | 0.003 | down |
| Sphingomyelins (SM) | 23 | 3 | ns | 0 | ns | 5 | ns |  |
| Cholesteryl Esters (Chol.Ester) | 26 | 0 | ns | 1 | ns | 2 | ns |  |

*p-value calculated from the sum of areas of each detected unique lipids of the same class. ns: non-significant
